# Deep Sequencing of RNA from Blood and Oral Swab Samples Reveals the Presence of Nucleic Acid from a Number of Pathogens in Patients with Acute Ebola Virus Disease and Is Consistent with Bacterial Translocation across the Gut

**DOI:** 10.1128/mSphereDirect.00325-17

**Published:** 2017-08-23

**Authors:** Miles W. Carroll, Sam Haldenby, Natasha Y. Rickett, Bernadett Pályi, Isabel Garcia-Dorival, Xuan Liu, Gary Barker, Joseph Akoi Bore, Fara Raymond Koundouno, E. Diane Williamson, Thomas R. Laws, Romy Kerber, Daouda Sissoko, Nóra Magyar, Antonino Di Caro, Mirella Biava, Tom E. Fletcher, Armand Sprecher, Lisa F. P. Ng, Laurent Rénia, N’faly Magassouba, Stephan Günther, Roman Wölfel, Kilian Stoecker, David A. Matthews, Julian A. Hiscox

**Affiliations:** a Public Health England, Porton Down, Salisbury, United Kingdom; b NIHR Health Protection Research Unit in Emerging and Zoonotic Infections, Porton Down, United Kingdom; c University of Southampton, South General Hospital, Southampton, United Kingdom; d Centre for Genomic Research Institute of Integrative Biology, University of Liverpool, Liverpool, United Kingdom; e Institute of Infection and Global Health, University of Liverpool, Liverpool, United Kingdom; f NIHR Health Protection Research Unit in Emerging and Zoonotic Infections, Liverpool, United Kingdom; g National Public Health Institute, National Biosafety Laboratory, Budapest, Hungary; h School of Biological Sciences, University of Bristol, Bristol, United Kingdom; i European Mobile Laboratory, Hamburg, Germany; j Institut National de Sante Publique, Conakry, Guinea; k Defence Science Technology Laboratories, Porton Down, United Kingdom; l Bernhard Nocht Institute for Tropical Medicine, Hamburg, Germany; m Bordeaux Hospital University Center, INSERM U1219, Bordeaux University, Bordeaux, France; n National Institute for Infectious Diseases, Lazzaro Spallanzani IRCCS, Rome, Italy; o Liverpool School of Tropical Medicine, Liverpool, United Kingdom; p Médecins Sans Frontières, Brussels, Belgium; q Singapore Immunology Network, Agency for Science, Technology and Research, Singapore; r Université Gamal Abdel Nasser de Conakry, Laboratoire Des Fievres Hemorragiques en Guinee, Conakry, Guinea; s Bundeswehr Institute of Microbiology, Munich, Germany; t School of Cellular and Molecular Medicine, University of Bristol, Bristol, United Kingdom; Boston University School of Medicine; Georgia State University; Centers for Disease Control and Prevention

**Keywords:** Ebola, Ebola virus disease, informatics, Plasmodium falciparum, RNA-seq, bioinformatics, coinfection, filovirus, gene expression, host-pathogen interactions, intracellular parasites, malaria

## Abstract

In this study, samples from the 2013–2016 West African Ebola virus outbreak from patients in Guinea with Ebola virus disease (EVD) were analyzed to discover and classify what other pathogens were present. Throat swabs were taken from deceased EVD patients, and peripheral blood samples were analyzed that had been taken from patients when they presented at the treatment center with acute illness. High-throughput RNA sequencing (RNA-seq) and bioinformatics were used to identify the potential microorganisms. This approach confirmed Ebola virus (EBOV) in all samples from patients diagnosed as acute positive for the virus by quantitative reverse transcription-PCR in deployed field laboratories. Nucleic acid mapping to *Plasmodium* was also used on the patient samples, confirming results obtained with an antigen-based rapid diagnostic test (RDT) conducted in the field laboratories. The data suggested that a high *Plasmodium* load, as determined by sequence read depth, was associated with mortality and influenced the host response, whereas a lower parasite load did not appear to affect outcome. The identifications of selected bacteria from throat swabs via RNA-seq were confirmed by culture. The data indicated that the potential pathogens identified in the blood samples were associated with translocation from the gut, suggesting the presence of bacteremia, which transcriptome data suggested may induce or aggravate the acute-phase response observed during EVD. Transcripts mapping to different viruses were also identified, including those indicative of lytic infections. The development of high-resolution analysis of samples from patients with EVD will help inform care pathways and the most appropriate general antimicrobial therapy to be used in a resource-poor setting.

**IMPORTANCE** Our results highlight the identification of an array of pathogens in the blood of patients with Ebola virus disease (EVD). This has not been done before, and the data have important implications for the treatment of patients with EVD, particularly considering antibiotic stewardship. We show that EVD patients who were also infected with *Plasmodium*, particularly at higher loads, had more adverse outcomes than patients with lower levels of *Plasmodium*. However, the presence of *Plasmodium* did not influence the innate immune response, and it is likely that the presence of EBOV dominated this response. Several viruses other than EBOV were identified, and bacteria associated with sepsis were also identified. These findings were indicative of bacterial translocation across the gut during the acute phase of EVD.

## Abstract (precis)

Our results highlight the identification of an array of pathogens in the blood of patients with Ebola virus disease (EVD). This has not been done before, and the data have important implications for the treatment of patients with EVD, particularly considering antibiotic stewardship. We show that EVD patients who were also infected with *Plasmodium*, particularly at higher loads, had more adverse outcomes than patients with lower levels of *Plasmodium*. However, the presence of *Plasmodium* did not influence the innate immune response, and it is likely that the presence of EBOV dominated this response. Several viruses other than EBOV were identified, and bacteria associated with sepsis were also identified. These findings were indicative of bacterial translocation across the gut during the acute phase of EVD.

## INTRODUCTION

The 2013–2016 Ebola virus (EBOV) crisis in West Africa devastated the health care and wider infrastructures of many communities, and subsequent sporadic cases associated with sexual transmission have been reported (1). The outbreak was unprecedented in scale, providing an opportunity for an in-depth analysis of infected humans and observations of previously unknown aspects of EBOV biology. The epidemic occurred at a time when high-resolution genomic analysis could be used to analyze samples obtained from patients during the outbreak (2–4). Using these approaches, viral genome evolution has been investigated (2), and the host immune response has been shown to play an important role in the outcome of infection (5). Additionally, differential cellular transcriptional profiles of blood samples from patients who presented at an Ebola virus treatment center have been shown to be associated with an outcome of either survival or death. Differences in activation of natural killer cells suggested a large acute-phase response and activation of the innate immune response (4).

Many of the patients from West Africa who had Ebola virus disease (EVD) would have had an underlying burden of infectious disease. Additionally, during an EVD breakout, other infections may be present and/or translocate from one organ to another, complicating disease and patient management and influencing infection outcome. Guinea, where the 2013–2016 outbreak originated, experiences high levels of malaria transmission (>1 case per 1,000 population), with Plasmodium falciparum being responsible for 100% of cases in 2013 (6). *Plasmodium* as a coinfection has been implicated in the outcome of EVD (7), and a cohort study suggested that the presence of *Plasmodium* was associated with survival (8). The presence of GB virus C (GBV-C) (formally known as hepatitis G virus) was examined in 49 patients positive for EBOV from Sierra Leone (9), where both survival and GBV-C status were found to be associated with age (9). There have been several case reports of Gram-negative septicemia in patients with EVD (10), although very little data exist regarding the frequency of this septicemia. However, the incidence of bacteremia appeared to be low upon clinic admittance (11). Assessing the presence of bacterial infection of the blood from large numbers of patients infected with high-consequence infections is complicated, particularly under field conditions of high containment, and the primary need is to provide an accurate diagnosis of EBOV.

During the outbreak in Guinea, the European Mobile Laboratory provided a frontline diagnostic service by testing blood samples for EBOV via quantitative reverse transcription-PCR (qRT-PCR) and for the presence of *Plasmodium* spp. by using an immunochromatographic assay (12). Patients who tested positive for EBOV were admitted into the Ebola Virus Treatment Centre and treated according to guidelines from Médecins Sans Frontières (13). These patients subsequently either went on to succumb to EVD (hospitalized fatalities) or survived infection (hospitalized survivors). The European Mobile Laboratory archived large numbers of these leftover diagnostic samples, primarily consisting of RNA. Additionally, oral throat swabs were taken from people in the community who died (community deaths) to test for EBOV. Blood samples were also taken from patients who were convalescent for EBOV to prove that these patients were EBOV negative and thus could be discharged from the Ebola Virus Treatment Centre.

A genomics approach was used to sequence nucleic acids present in the samples and to map reads to microorganisms (not mapping to the human genome). Identification of transcripts to pathogens would be indicative of other microorganisms potentially present in the blood of patients with EVD, indicating translocation from the gut during the acute phase and reflecting underlying burdens of disease. The data indicated that the genomics pipeline successfully identified and quantified the amount of EBOV. The data suggested that the presence of *Plasmodium* had been underreported based on the antigen capture test and highlighted how a genomics approach could provide additional evidence of this parasite and connect it to an identified patient sample—particularly for retrospective analyses. The study found that most patients with acute illness on admittance to the Ebola Virus Treatment Centre already had extensive evidence in their blood of nucleic acid mapping to bacteria (the blood should have been effectively sterile), as well as nucleic acid mapping to viruses and parasites. The types of microbes identified argued strongly for bacterial translocation across the gut wall. These results have implications for the design of supportive care platforms, including empirical antimicrobial use.

## RESULTS

**FIG 1:**
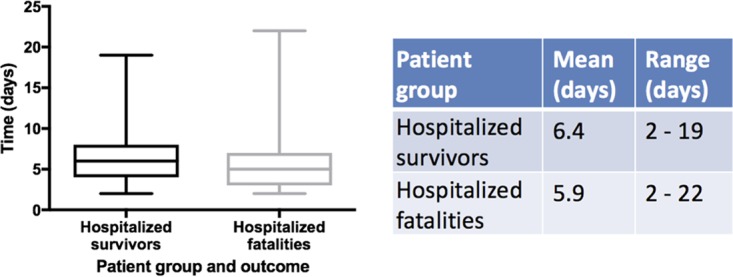
Mean time between symptom onset and sample procurement for two of the three different patient categories, hospitalized survivors and hospitalized fatalities.

A genomics approach was used to determine whether other microorganisms were present in samples from patients who tested positive for EBOV. To do this and to optimize the approach, several different types of patient samples were used (Table 1). These focused on oral swabs taken from people who had died in the community and blood samples that were taken as diagnostic samples when patients presented at an Ebola Virus Treatment Centre in Guinea. These patient cohorts were classified as hospitalized fatalities and hospitalized survivors, depending on their outcome after the sample was taken. All of these hospitalized patients in the study had acute EVD at the time of sampling. The mean time between symptom onset (as reported by the patient) was not significantly different between the two cohorts (Fig. 1).

**TABLE 1:** Summary details of patients from which RNA-seq data were selected for this study^a^

Patient trait	Community deaths	Hospitalized fatalities	Hospitalized survivors	Convalescent controls
*n*	24	118	44	16
% male	29.1	40.1	29.5	93.75
Age range	1–70 yr	2 mo–80 yr	10 mo–68 yr	18–40 yr
Mean age (yrs)	33.35	28.44	33.10	30
				
EBOV *C*_*T*_				
Range	12.37–31.85	12.08–26.55	15.77–31.60	NA
Mean	18.81	17.23	21.67	NA

a Samples with similar threshold cycle (*C*_*T*_) values were chosen, as this would indicate similar viral loads. It was not possible to directly correlate *C*_*T*_ values to viral load. NA, not applicable.

Blood samples were also taken from EVD patients convalescing from the disease who were confirmed EBOV negative by qRT-PCR. The latter group acted as a control (uninfected group). The host blood transcriptome from this healthy convalescent group was not significantly different from a healthy control group of patients (4) who were located in British Columbia (14). These healthy patients were temporally and spatially distinct from the outbreak, indicating that the host blood transcriptomes from convalescent and healthy controls were equivalent (4). We noted, however, that the collection of blood from the convalescent patients would have been in a more controlled environment than the collection of blood from the patients with acute EVD.

### Identifying the total spectrum of microbes present in the blood samples.

Rather than first trying to identify separate pathogens, based on predetermined lists of pathogens that could be potentially present in blood samples taken from individual patients, an unbiased search approach was taken. Sequencing data from all the acute patients with EVD and convalescent patients were pooled to first determine which pathogens were present, using a combination of *de novo* and reference-based mapping approaches. The identified pathogens were then used to form a specific query database at the individual patient level to determine which pathogens were present in which samples, thus reducing computational time.

**FIG 2:**
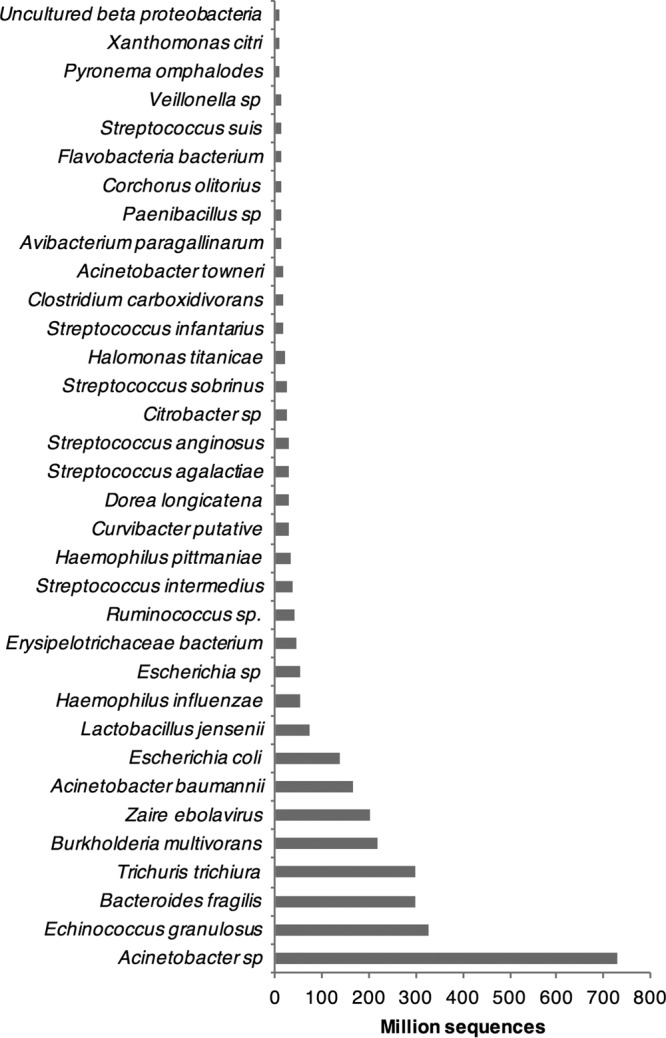
Illustrative examples for the total number of reads mapped to species identified in the blood samples from patients with acute EVD.

Sequence reads were combined from all the patients in these groups and any that aligned to the human genome were removed from the analysis, leaving approximately 1 TB of fastq data. Next, these reads were processed by using the Trinity read normalization software before assembling into ~1.2 million contigs using Trinity. These contigs were searched in BLAST using Diamond BLAST to find the nearest homologues and then they were manually searched to confirm the presence of the EBOV genome. As expected, a near-full-length genome corresponding to EBOV was assembled by use of Trinity, allowing calibration and verification of the approach. In addition, evidence of genetic material belonging to other pathogens was identified (provided as a fully searchable Excel file in Table S1 in the supplemental material and abstracted in Fig. 2).

10.1128/mSphereDirect.00325-17.2 TABLE S1 Evidence of genetic material belonging to other pathogens. Download TABLE S1, XLS file, 12.5 MB. Copyright © 2017 Carroll et al. 2017 Carroll et al. This content is distributed under the terms of the Creative Commons Attribution 4.0 International license.

Examples are also presented in Table 2 (for bacterial, fungal, and parasitic pathogens) and Table 3 (for viral pathogens) to illustrate the mix of microorganisms and gene products identified. The largest number of reads mapped to *Acinetobacter* spp. (prevalent in intensive care units), *Echinococcus granulosus* (a tape worm), Bacteroides fragilis (a commensal bacterium associated with the normal flora of the colon), Trichuris trichiura (a whipworm normally found in the intestine), and Burkholderia multivorans (a bacterium associated with chronic infections in cystic fibrosis), followed by EBOV. The data immediately illustrated the potential complexity of microorganisms in acutely ill patients who are positive for EBOV and that these other organisms may potentially confound treatment. Obvious from this effort was the identification of sequence reads mapping to Plasmodium falciparum, the causative agent of malaria tropica. The presence of this parasite was also confirmed by an antigen-based approach (a rapid diagnostic test [RDT]) for many of the samples in the European Mobile Laboratory deployed in Guinea.

**TABLE 2:** Exemplary data on the variety of microorganisms identified in pooled RNA-seq data from 179 patients positive for EBOV based on qRT-PCR

Organism	Protein	% identity	Alignment	E value
Escherichia coli G58-1	Glycosyl transferase family 2	100	583	0.00E+0
Lactobacillus fermentum	DNA-directed RNA polymerase subunit beta	100	722	0.00E+0
Wohlfahrtiimonas chitiniclastica	Hypothetical protein, partial	100	583	0.00E+0
Plasmodium falciparum UGT5.1	Hypothetical protein C923_00328	100	511	4.50E−298
Candida albicans WO-1	Acetyl coenzyme A carboxylase	100	504	2.50E−293
Streptococcus pneumoniae EU-NP04	Transposase DDE domain protein	100	493	2.90E−292
*Myroides* spp.	GMP synthase	100	508	3.20E−292
Haemophilus influenzae	Phosphoribosylformylglycinamidine synthase	100	500	1.80E−289
Acinetobacter baumannii AB307-0294	2-Isopropylmalate synthase	100	479	1.20E−276
Providencia rettgeri DSM 1131	DNA polymerase III subunit alpha	100	483	9.70E−276
Plasmodium falciparum Vietnam Oak-Knoll	Hypothetical protein PFFVO_04502	100	476	2.20E−270
Ureaplasma urealyticum	Cell division protein FtsH	100	478	8.20E−264
Lactobacillus fermentum	6-Phosphogluconate dehydrogenase	100	457	3.70E−260
Plasmodium falciparum Tanzania (2000708)	Hypothetical protein PFTANZ_03315	100	450	5.70E−260
Streptococcus sanguinis	Collagen-binding protein	100	439	2.80E−251
Acinetobacter baumannii	Putrescine/spermidine ABC transporter	100	444	5.10E−251
Haemophilus haemolyticus	Catalase	100	415	8.40E−250
Plasmodium falciparum 3D7	Elongation factor 1 alpha	100	433	4.60E−249
Plasmodium falciparum MaliPS096_E11	Histidine-tRNA ligase	100	403	1.10E−235
*Myroides* spp.	Multispecies collagenase	100	413	6.20E−232
Plasmodium falciparum Tanzania (2000708)	ATP-dependent protease HslVU, ATPase subunit	100	410	2.10E−229
*Campylobacter* spp.	Multispecies GTP-binding protein	100	407	5.10E−228
Streptococcus parasanguinis	Amino acid transporter	100	402	8.80E−228
Haemophilus parainfluenzae	l,d-Transpeptidase	100	393	3.80E−224
Escherichia coli	Leucyl-tRNA synthetase	100	383	9.40E−224
*Myroides* spp.	Diaminopimelate decarboxylase	100	388	9.30E−223
Myroides odoratimimus	TonB-dependent receptor	100	389	1.80E−222

**TABLE 3:** Virus sequences identified in pooled RNA-seq data from 179 patients positive for EBOV based on qRT-PCR and clinical diagnosis

Virus	Protein	% identity	Amino acid length	E value
EBOV	L protein	96.8	2,212	0.00E+0
GB-C	Polyprotein	99.1	328	1.50E−190
				
HHV-4	BALF2	100	100	1.9E−51
	LF2 protein	100	166	4.9E−91
	BALF5	98.8	172	4.4E−88
	BALF4	100	257	6.6E−142
	DNA polymerase catalytic subunit	99.2	131	2.3E−71
	BHRF1	100	121	7.4E-66
	BARF0	99.2	120	6.5E−62
	Early antigen D	100	118	2E−60
	gp110 precursor	100	95	1.3E−49
	A73 protein	100	94	6.1E−50
	LF1	98.9	92	1.1E−48
	Putative BHLF1 protein	98.7	75	4.8E−42
	BFRF1	100	71	4.1E−32
	BMRF2	100	68	7.6E−31
	dUTPase	98.5	68	3E−32
	BFRF2	100	67	4.2E−32
	LF3 protein	100	45	0.0000000000000002
	K15	100	37	0.00000000000056
				
HHV-5 (cytomegalovirus)	IRL4	100	107	3.1E−53
	IRL3	94.1	101	1.20E−46
	IRL7	100	82	1.60E−37
	RL5A	63.6	66	9.30E−15
	IRL5	55.1	69	3.50E−12
	UL109	68.1	47	2.10E−10
				
Hepatitis A virus	Polyprotein	100	91	5.5E−45
Hepatitis B virus	Polymerase	92.8	503	3.30E−278
Pepino mosaic virus	Replicase	99.4	163	4.60E−87
Sewage-associated gemycircularvirus-4	Replication-associated protein	100	105	3.60E−57
				
Rotavirus A	VP6	98.8	86	8.1E−41
	VP1	100	75	1.1E−33
	NSP3	169	251	1.5E−21
				
Papillomavirus	Minor structural protein- interacting protein	100	56	3.40E−24
	L2	97.2	72	1.50E−34
	Regulatory protein	98.5	67	5E−30
				
Torque Teno virus 20	Unnamed protein product	100	32	9.10E−9
				
Tobacco mosaic virus	Replicase	97.2	1,521	0.00E+0
	Capsid protein	100	102	7.2E−49
	126-kDa protein	99	314	2.30E−181
	Movement protein	97.4	268	8.60E−145

### The convalescent control group samples provided a control for potential environmental contamination for blood sampling and the informatics assignment pipeline.

**FIG 3:**
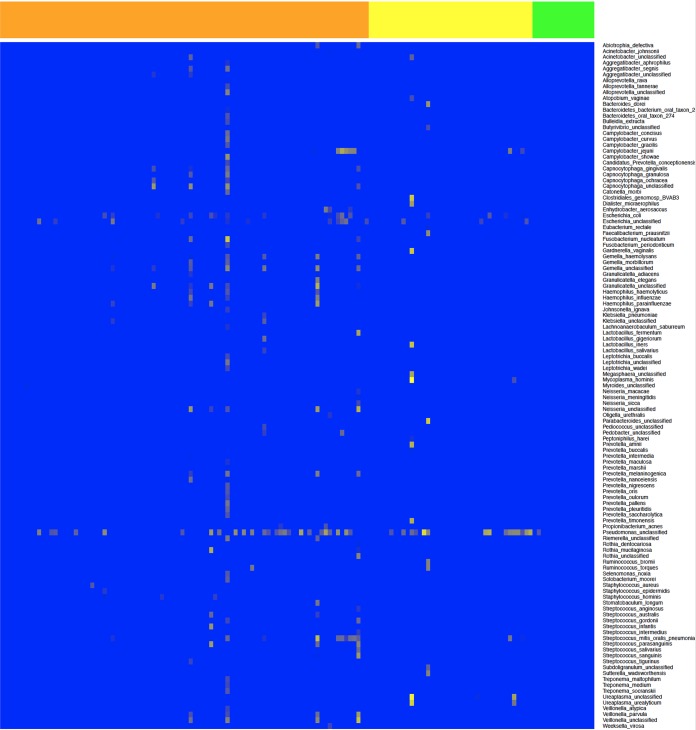
Heat map showing the bacterial species (*y* axis) identified by mapping to nucleic acids by comparing all patient cohorts who had a blood sample taken when they had acute EVD. The *x* axis shows individual patients from the convalescent control group (green) (*n* = 16), hospitalized survivors group (yellow) (*n* = 44), and hospitalized fatalities group (orange) (*n* = 118); data for these groups are also separated by vertical lines. The colors represent the arbitrary read depth of the sequence, with yellow indicating a greater sequence read depth.

The convalescent control group provided a means to test the high-throughput RNA sequencing (RNA-seq) and bioinformatics approaches in terms of sample handling and potential environmental contamination during the stages involved in obtaining samples from patients, sample processing, and transport, all the way through to sequencing. The prediction was that in the convalescent group of patients that had been shown to EBOV negative by qRT-PCR and *Plasmodium* negative through RDT, these and other organisms should not have been found, as blood is effectively sterile. In this group of 16 patients, no transcripts mapping to Plasmodium falciparum or to EBOV were identified. In only one patient were transcripts identified that mapped to a bacterium, which was identified as *Pseudomonas* at the species level (Fig. 3). These results thus suggest that any potential environmental contamination was minimal and the informatics pipeline did not identify spurious “hits.”

### Defining the oral microbiome and selected confirmation by mass spectrometry.

**FIG 4:**
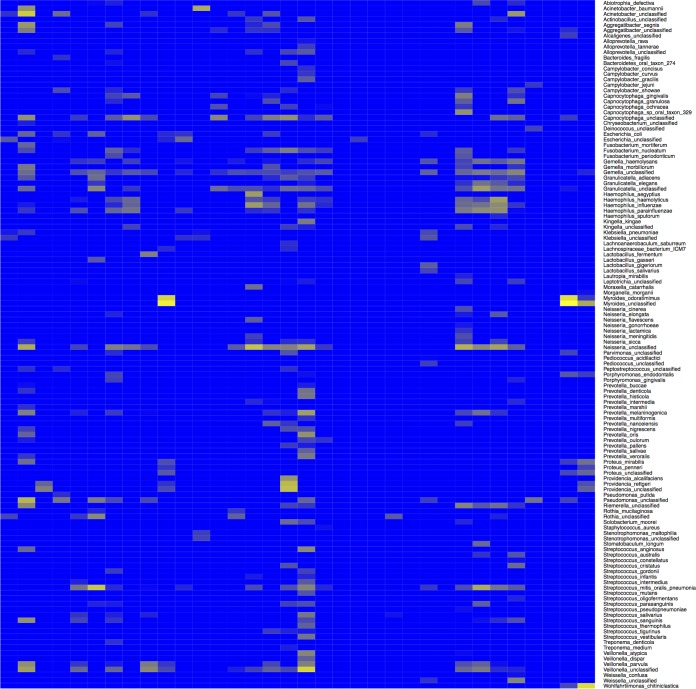
Heat map showing the bacterial species (*y* axis) identified mapping to nucleic acids in a swab sample taken from an individual patient. All patients in this category were deceased at the time of sampling (*n* = 24). The color represents the arbitrary read depth of the sequence, with yellow indicating a greater sequence read depth.

Sequence analysis of the RNA prepared from swabs taken from community deaths (*n* = 24) illustrated a range of nucleic acids mapping to bacteria that would be expected in the oral microbiome. This included sequence reads mapping to Neisseria meningitidis, a Gram-negative bacterium that can cause sepsis (Fig. 4). A mass spectrometry-based approach was then used to confirm the identification of individual bacterial species in the oral microbiome of patients in the community death cohort. Oral samples were cultured at high containment facilities, and colonies were recovered from the throat swabs of five separate patients in the community death group; these samples tested positive for bacteria and fungi by mass spectrometry (Table 4). A score for the matrix-assisted laser desorption ionization–time of flight (MALDI-TOF) results between 2.000 and 2.2999 indicated secure genus identification, and scores between 2.300 and 3.000 indicated a high-probability species identification. Identification of bacteria by mass spectrometry matched those identified by the RNA-seq and computational approaches. For example, Proteus mirabilis was identified by RNA-seq and by mass spectrometry from swabs taken from two patients, and Rothia mucilaginosa and Streptococcus salivarius were identified in individual patients.

**TABLE 4:** Details of bacterial isolates from throat swab samples identified by mass spectrometry approaches

EBOV *C*_*T*_	Age	Sex	MALDI-TOF result(s)^a^
24	22 yr	Male	Proteus mirabilis (2.537)
19	33 yr	Female	Rothia mucilaginosa (2.181)
25	60 yr	ND^b^	Proteus mirabilis (2.427)
14	4 mo	Male	Staphylococcus aureus (2.378) Acinetobacter baylyi (2.054)
26	63 yr	Male	Streptococcus salivarius (2.117) Candida albicans (2.141)

a Scoring for MALDI-TOF results was as follows: 3.000 to 2.300, highly probable species identification; 2.2999 to 2.000, secure genus identification.

b ND, no data.

### Identification of transcripts mapping to bacteria in the blood of EVD patients was indicative of bacterial translocation from the gut.

Nucleic acids from both bacteria (Table 2; Fig. 3) and fungi (Table 2) were identified in the blood from EBOV-positive patients presenting at the Treatment Centre and testing positive for EBOV. Confirmatory blood cultures were attempted at high containment but were not successful, possibly due to multiple freeze-thaw cycles and suboptimal sample transport. Although it was problematic to distinguish what may be commensal microorganisms from these data, nevertheless, several bacterial species were identified, based on sequence reads mapped, that have been associated with disease in humans, particularly sepsis (15). These species included Acinetobacter baumannii, which thrives in individuals with weakened immune systems and is considered a nosocomial agent and has multidrug resistance (16). Streptococcus pneumoniae and Haemophilus influenzae are found asymptomatically in the upper airways of asymptomatic healthy carriers, but if found in the bloodstream, both pathogens can cause pneumonia, meningitis, and sepsis. Haemophilus influenzae was identified in both the blood (and swab) samples. Several patients had evidence of a wide range of bacteria, based on nucleic acid sequences identified, including one patient in the hospitalized survivors category. This patient had evidence of nucleic acids that mapped to bacteria associated with sexual transmission and vaginal infection, including Gardnerella vaginalis, Lactobacillus iners, and Ureaplasma urealyticum. Nucleic acids from *Enterobacteriaceae* was also identified in many patients. These are the predominant pathogenic bacteria in the bowel, where bacterial translocation would be expected to originate in EVD. Examples included sequence reads mapping to Gram-negative bacteria such as Escherichia coli, *Klebsiella* spp., *Enterobacter* spp., *Shigella* spp., *Salmonella* spp., *Pseudomonas* spp., and Gram-positive bacteria such as *Enterococcus* spp. Therefore, the data indicate that most patients with EVD sequenced in this study had evidence of bacterial translocation across the gut.

### The effect of bacteria on the host response in EVD patients.

**FIG 5:**
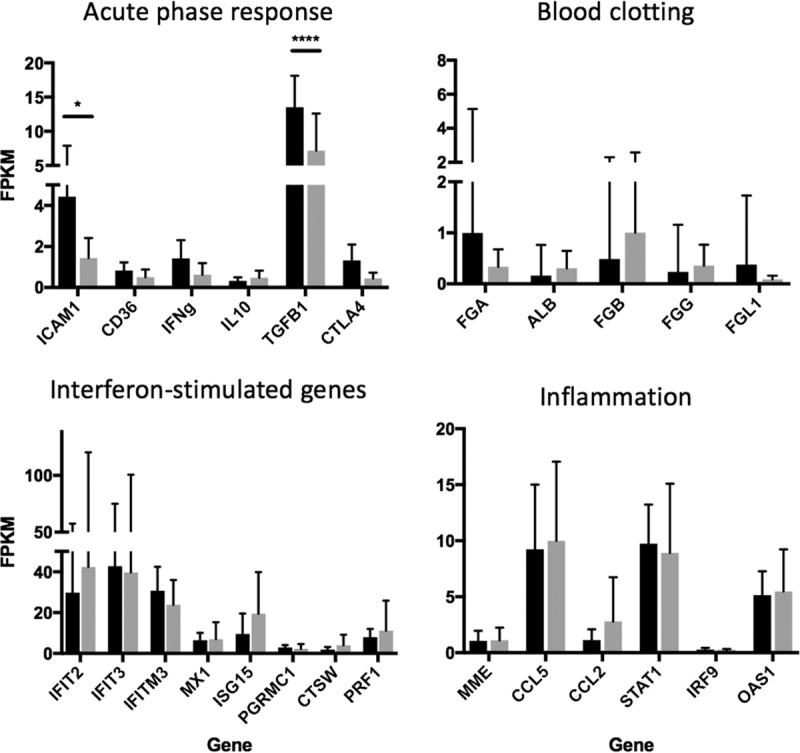
Relative abundance of transcripts mapping to host transcripts associated with pathways involved in the acute-phase response, blood clotting, innate immune response, and inflammation in patients with EVD (positive by RNA-seq and qRT-PCR) who were confirmed positive (*n* = 22; gray) or negative (*n* = 9; black) for at least one bacterial coinfection (by RNA-seq). Relative gene expression levels were measured as fragments per kilobase of transcript per million mapped reads (FPKM). Each of the graphs presented is based on the average FPKM values. Statistical analysis was performed using a two-way ANOVA with Sidak’s multiple-comparisons adjustment (Prism 7; GraphPad, CA).

Analysis of the host response in peripheral blood samples from patients with acute EVD identified several differences compared to healthy controls (4). There were also differences in the host transcriptomes between patients with EVD who survived versus those who succumbed to EVD (4). These included pathways involved in the innate response, blood coagulation, and the acute-phase response (4). To investigate whether the potential presence of bacteria in the blood of patients with EVD influenced the host response, transcriptome changes in these exemplary pathways were compared between the different groups of patients. This analysis made use of matching host transcriptome data that had been determined for this group of patients (4). The abundance of transcripts mapping to selected genes were chosen to assess the effect of pathogen burden on the different exemplary pathways. The data indicated that certain transcripts associated with the acute-phase response (intercellular adhesion molecule 1 [ICAM-1] and transforming growth factor β1 [TGF-β1]) were present at different abundance levels in EVD patients with versus without sequence reads mapping to bacteria in their blood (Fig. 5). In contrast, there were no significant differences in the abundance levels of selected transcripts associated with blood clotting, interferon (IFN) stimulation, or inflammation, at least in the limited sample sets used in this study.

### Analysis of *Plasmodium* and correlation with outcome.

Matching sequencing reads to the *Alveolata* superphylum from the blood samples suggested that most transcripts corresponded to P. falciparum (Fig. S1), and on a per-patient basis, the presence of the malaria parasite was confirmed by RDT. The data indicated that 84% of blood and plasma samples contained sequence reads mapping to P. falciparum transcripts. However, these sequence reads varied from an FPKM (a measure of sequence read depth and abundance of RNA transcripts mapping to P. falciparum) of 59, in a sample from a patient with acute EVD with the lowest number of sequences, to an FPKM of 358820, representing the highest number of sequence reads mapping to *Plasmodium* spp*.*

10.1128/mSphereDirect.00325-17.1 FIG S1 Phylogenetic tree showing alveolates identified through Diamond BLASTX querying of Trinity-assembled contigs against the nonredundant NCBI database. An E value cutoff of 1 × 10^−10^ was applied with no additional filtering. *Plasmodium* species are indicated with red branches, and yellow and red bars indicate the relative abundance levels of sequences associated with species, based on the length of BLAST hits; yellow branches represent log values and red branches represent linear values. Many of the BLAST hits to species with apparently very low hit lengths may have been artifactual but have been retained to provide a contextual background. Download FIG S1, PDF file, 0.4 MB. Copyright © 2017 Carroll et al. 2017 Carroll et al. This content is distributed under the terms of the Creative Commons Attribution 4.0 International license.

**FIG 6:**
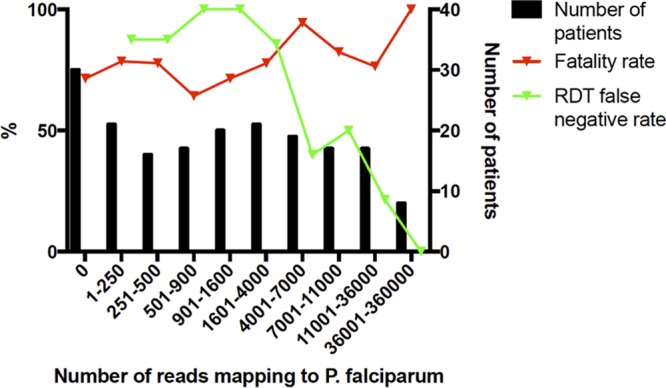
Effect of an increased number of reads mapping to P. falciparum. The green line, associated with the right-hand *y* axis, shows the proportion of patients who tested negative for malaria according to the RDT but for whom reads to P. falciparum were detected during RNA-seq analysis. The red line, also associated with the right-hand *y* axis, shows the patient fatality rate according to different levels of reads to P. falciparum. The left-hand *y* axis shows the abundance of patients in each bin, i.e., the number of individuals who were grouped into each category. The graph was created using Prism (GraphPad, CA).

Comparison with the RDT results suggested that the presence of P. falciparum may have been underrepresented in the sampled patient population (Fig. 6). The data were then analyzed to investigate whether the P. falciparum load influenced the outcome of EVD (Fig. 6). As the RDT is qualitative, sequence read depth was used as a proxy measurement for the amount of *Plasmodium* present, and these results are akin to those from qRT-PCR, which is used as a confirmatory technique for the laboratory diagnosis of malaria (17, 18). Here, sequence read depths were divided into arbitrary bins based on approximately the same numbers of patients. Of the patients experiencing the highest reads mapping to P. falciparum, 87% succumbed to a fatal infection (*n* = 6). However, while the data indicated that in general mortality increased with sequence read depth, there was no absolute correlation, at least at the sequence read depths measured in this analysis.

### The effect of *P. falciparium* on the host response in EVD patients.

**FIG 7:**
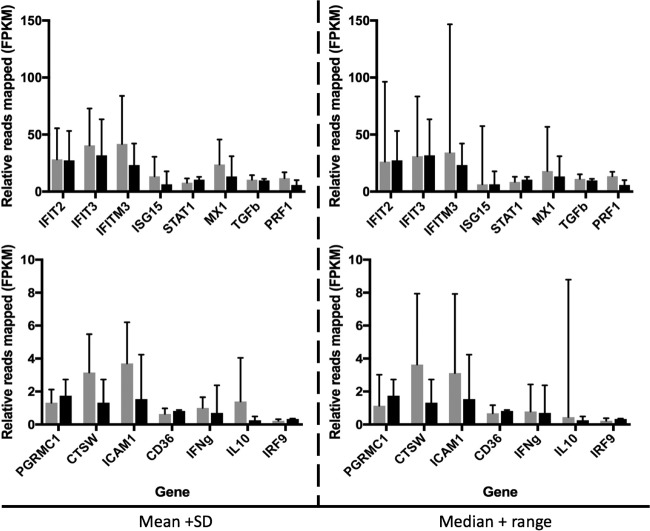
Relative abundance of transcripts mapping to host transcripts associated with malaria and the innate immune response in patients with EVD who were confirmed positive (gray; *n* = 10) or negative (black; *n* = 13) for P. falciparum (by RNA-seq and RDT). Relative gene expression levels were measured as the fragments per kilobase of transcript per million mapped reads (FPKM). Each set of graphs (left and right) is based on average FPKM values. Two analyses of the data are presented, and means and standard deviations (left) or medians and total ranges (right) are shown.

**FIG 8:**
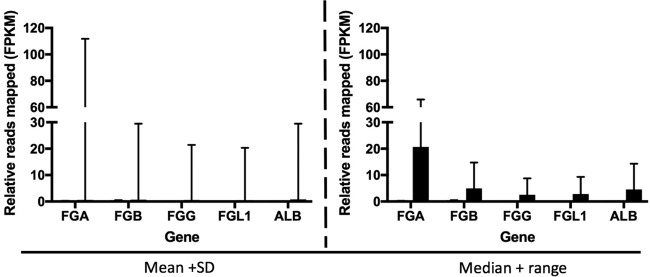
Relative abundance of transcripts mapping to host transcripts associated with coagulation in patients with EVD and confirmed positive (gray; *n* = 10) or negative (black; *n* = 12) for P. falciparum (by RNA-seq and RDT). Relative gene expression levels were measured as fragments per kilobase of transcript per million mapped reads (FPKM). The graphs are based on average FPKM values. Two analyses of the data are presented, showing the means and standard deviations (left) and medians and total ranges (right).

For EVD patients with the highest depth of sequence mapping to P. falciparum, the host response may have been different for patients infected only with EBOV. To investigate this, the abundance of transcripts corresponding to markers of innate immunity and associated with malaria tropica were compared. The two groups of patients selected were patients with EBOV that were positive for P. falciparum, determined by both RNA-seq and RDT and using data from patients with the highest mapped reads to P. falciparum (*n* = 10), versus patients with EBOV but for whom both RDT and sequence read results were negative for P. falciparum (*n* = 13). This analysis made use of matching host transcriptome data that had been determined for this group of patients (4). Selected genes were chosen whose abundance levels had previously been associated with severe malaria tropica in the absence of EBOV infection (IFIT2, IFIT3, IFITM3, ISG15, STAT1, MX1, TGF-β, PRF1, PGRMC1, CTSW, ICAM-1, CD36, IFN-γ, interleukin-10, and IFN regulatory factor 9) (19–22). The abundance levels of the gene transcripts were compared, and these data are presented in two ways (Fig. 7). The first way shows the mean value plus the standard deviation for each transcript. The second way shows the median value plus the total range; the latter analysis is additionally presented because the transcript abundance in individual patients did not follow a normal distribution. The data indicated no significant differences in the abundance of transcripts associated with the innate response between patients who were EBOV positive and P. falciparum negative compared to patients who were positive for both EBOV and P. falciparum. This suggested that P. falciparum has no additional effect on the innate response. A similar analysis was performed on the abundance of transcripts associated with the coagulation pathway (FGA, FGB, FGG, FGL1, and ALB) (Fig. 8). These were the most differentially regulated transcripts between the EVD and control groups (4). The data indicated that transcripts associated with the coagulation pathway were increased in abundance in several of the EVD patients who tested negative for P. falciparum, compared to EVD patients who tested positive for P. falciparum. Note that this analysis was based on a limited patient sample size (23 in total) due to the stringent inclusion criteria used.

### Nucleic acids from viruses were identified in the blood of patients with EVD.

**FIG 9:**
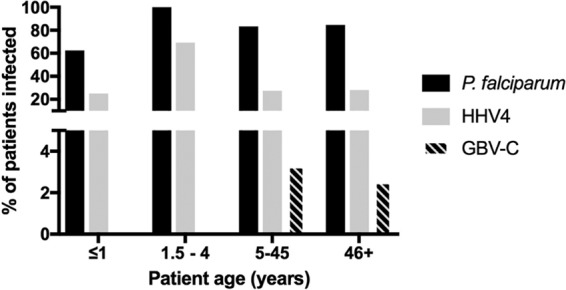
The presence of nucleic acids from certain viruses varies with patient age. Patients were grouped according to their immunological maturity (≤1 year [*n* = 8], 1.5 to 4 years [*n* = 13], 5 to 45 years [*n* = 120], and 46+ years [*n* = 39]), and the proportions of each group with reads mapping to P. falciparum, HHV4, and GBV-C are shown.

Nucleic acid mapping to several different viruses were identified in the blood of EVD patients. These included GB virus C, human herpesvirus 4 (HHV4, or Epstein-Barr virus), human herpesvirus 5 (cytomegalovirus), and rotavirus. The latter virus is associated with gastroenteritis and may be present in the blood through gastrointestinal bleeding (23) and has been associated with extraintestinal disease (24). No evidence was found in the samples for common circulating respiratory viruses. Data indicated that in approximately 30% of the samples, nucleic acid mapping to HHV4 was identified. The identification of BALF2 mRNA transcript from HHV4 (Table 3) implies there was HHV4 activation in EVD patients (25). In contrast to a previously report (9), in our patient sample set we found no correlation between the presence of nucleic acid from GBV-C virus and survival. However, our sample population included only 5 patients identified to have nucleic acid from GBV-C, with only 3 of these samples have been obtained during acute infection with EBOV. Nucleic acid mapping to HHV4 was most prevalent in the 1- to 4-years age group category (as was P. falciparum), whereas nucleic acid mapping to GBV-C virus appeared more prevalent in older age groups (Fig. 9). Somewhat surprisingly, several plant virus genomes/transcripts were also identified, with the highest and most confident hit being tobacco mosaic virus (TMV), for which the full genome was recovered. Our review of the literature suggested that serum antibodies to TMV can be identified in humans (26). Taken at face value, our data set suggest that plant virus genomes (and, by implication, plant viruses) can be found in blood, and this finding again may be indicative of a breakdown in the integrity of the gut wall. However, environmental contamination cannot be ruled out, especially during sample gathering. However, we note that at least for sequencing, none of the staff was cigarette or cigar smokers, and no plants were grown in the laboratory area.

## DISCUSSION

This study investigated the presence of transcripts from viral, bacterial, fungal, and parasitic pathogens in patients infected with EBOV. The study made use of samples taken for routine diagnostic testing for EVD. This is the first systematic analysis of pathogens in patients infected with EBOV based on an unbiased deep sequencing approach. This approach did not rely on specific amplification of pathogens in selected growth media or enrichment of genomic sequences (e.g., by RT-PCR or PCR). Samples included those taken from patients acutely ill with laboratory-confirmed EVD infection prior to being admitted to the Ebola Virus Treatment Centre. Most of these patients were admitted around 6 days into the illness (based on when the patient thought they started feeling unwell) (Fig. 1). This was still early in the disease course, but based on clinical studies the majority of patients would be predicted to have entered the gastrointestinal stage of illness (which occurs after 3 days [27, 28]), and therefore may also be predicted to have bacterial translocation from the gut to the blood. Blood samples taken from convalescent patients were used as a control to establish background levels of transcripts mapping to pathogens. As noted, the taking of blood from this group of patients would have been different from that from patients with acute EVD. This may have had an impact on which microbes were present, although we note that there was no one uniform microbial signature in the blood from patients with acute EVD. In the main, blood samples were used for the analysis presented herein, although some plasma sample results were also incorporated. There may be subtle differences in the transcriptome mapping to nucleic acids from pathogens in these samples, as sequence reads from blood would map to both extra- and intracellular pathogens, whereas with plasma, sequence reads would be predicted to map to extracellular pathogens.

The presence of other microorganisms can have a significant impact on the morbidity and mortality associated with primary infection. In a recent study, parasitemia (not clinical malaria) was positively associated with survival in patients with EVD (8). This study used PCR-based approaches to identify the presence of P. falciparum. Data from the analysis of samples gathered by the European Mobile Laboratory suggested that high parasite load (as determined by RDT, which likely indicates clinical malaria) corresponded with a poor outcome in children between 5 and 14 years old (12). The deep sequencing analysis suggested a larger proportion of patients with EVD had sequence reads mapping to P. falciparum than was recorded by use of RDT. P. falciparum RDTs have a limited sensitivity and therefore mainly detect patients with higher levels of parasites in the blood, as found with acute malaria disease. Molecular techniques have shown a high background prevalence of *Plasmodium* (29).

The effect of *Plasmodium* on patient outcome with this data set varied according to the method by which *Plasmodium* status was determined (Fig. 6). Via the RDT, *Plasmodium* appeared to impact patient outcome (*P* = 0.053; analysis of variance [ANOVA] performed using software from SPSS, Inc.). However, this effect was eliminated once P. falciparum status was confirmed using RNA sequencing data. This could be because with RNA-seq a larger proportion of individuals in the sample population tested positive for P. falciparum: 84% (156 out of 186) compared to 33% (40 out of 121) by RDT. The discrepancy between RDT and RNA-seq data was reduced as the number of reads to *Plasmodium* spp. increased. The RDT detects patients with high parasitemia and misses patients with low parasite levels. Thus, it is not surprising that a high *Plasmodium* spp. load has an impact on patient outcome, whereas a low *Plasmodium* load has a less pronounced effect or no impact. Individuals with a high *Plasmodium* load are likely to have been sampled at the time they were experiencing the acute phase of the infection. Acute-phase infections are associated with strong systemic proinflammatory responses (30), which are known to have antiviral effects. One hypothesis is that these responses may also act synergistically with virus-induced immunopathogenic responses. People with a low parasite load load are likely to harbor a chronic asymptomatic infection, which is characterized by mild or no inflammation and thus might be predicted not to interfere with Ebola virus infection. This was investigated by comparing the abundance of gene transcripts associated with the innate response stimulated by P. falciparum in EBOV-positive patients who had the highest sequence read depth mapping to P. falciparum and were also RDT positive, versus EBOV-positive patients who had no reads mapping to P. falciparum and were also RDT negative. The analysis made use of matched transcriptomic data to investigate the host response to EBOV infection (4). Overall, there was no significant difference in the abundance of gene transcripts associated with the innate response (Fig. 7), but there were potential differences in the abundance of transcripts associated with blood coagulation (Fig. 8). We note, however, that this analysis was based on a limited number of patients with matched host transcriptome data.

Nucleic acids from several bacterial species were identified in the blood and plasma samples from patients with EVD (Fig. 3). Sequence reads mapping to multiple organisms were identified in the patient samples, for example, *Prevotella* a genus of Gram-negative bacteria. Several studies have found that *Prevotella* dominates the African gut microbiome (31, 32), giving credence to the hypothesis of the translocation of bacteria from the gut to the blood. However, across both hospitalized survivors and hospitalized fatalities, in a minority of patients there was no evidence of nucleic acids from bacteria, at least at the sequence read depths used in this study. Additionally, the approach did not provide antimicrobial sensitivity or clear identification of dominant causative organisms in a polymicrobial picture. The use of data generated by mapped reads on bacterial identification should be treated with some caution, due to the close phylogenetic relationships between nonpathogenic and pathogenic representatives of a species. However, many of the identified bacteria are associated with sepsis, and consideration should be given to appropriate antibiotic therapy. Clinical guidelines suggest empirical antibiotic treatment for patients with EVD who often present with nonspecific symptoms and signs that are difficult to distinguish from malaria, typhoid fever, or bacterial sepsis (33). The rationale for empirical antibiotic therapy in EVD is 2-fold. First, it is used to provide broad-spectrum antibiotic cover for suspected EVD patient who have a range of infectious diseases, recognizing the lack of diagnostic capacity and resources in Ebola Treatment Centres. Second, it is to provide empirical Gram-negative/anaerobic antibiotic cover for patients with confirmed EVD who are at risk of secondary bacteremia, which is thought to originate from translocation across the bowel wall that is associated with the gastrointestinal clinical stage of EVD. Most patients showed evidence of nucleic acids from *Pseudomonas* in their blood, and supportive antibiotic therapy should take into account the possible presence of this bacterial species.

Unsurprisingly, oral swab samples from community deaths indicated bacterial diversity and bacterial load and were included for completeness (Fig. 4). This technique became routinely utilized in standard postmortem pathological sampling during this outbreak, with recognized limitations. The bacteria present in this sample set, and their growth, may be reflective of agonal processes or may represent growth postmortem. These samples included Wohlfahrtiimonas chitiniclastica, which was likely deposited by flies postmortem, and Neisseria meningitidis, a causative agent of meningitis, which was identified in both blood samples and swabs. Mass spectrometry also confirmed the identification of some of the species in throat swabs, thus validating the sequencing and bioinformatics approaches. Note that it was not possible to directly compare a throat swab with a blood sample from the same patient. Throat swabs were only used in the community deaths category, and only blood samples were taken when the patient presented at the treatment center. The species identified by mass spectrometry included bacteria and fungi. These microorganisms can be found as transitional flora in the upper respiratory tract. However, in case of a weakened immune system, these opportunistic pathogens may cause bacterial sepsis, pneumonia, or bacteremia. The successfully cultured bacteria are likely to constitute only a small part of the real bacterial flora, but many of the bacteria and fungi cannot be cultured due to the long storage time at −80°C and repeated freeze-thaw cycles. This illustrates the versatility of the sequencing pipeline that only requires RNA, returning large overlapping data sets and not requiring multiple steps at high containment.

Nucleic acids from several viruses were identified that correlated with the age of the patient, such as HHV4 (Epstein-Barr virus) (Fig. 9). No evidence was found to suggest that, of the viruses potentially identified, there was a correlation with outcome or that any virus had a synergistic effect. One transcript corresponding to the envelope glycoprotein of human immunodeficiency virus 1 (HIV-1) was found (Table S1), but no other HIV-1 transcripts were identified. The prevalence of adult HIV-1 in Guinea is 1.7% (2012 estimate). Therefore, in the patient cohort examined in this study, we would have predicted approximately 3 cases. However, if the virus was present in low copy number, then possibly higher sequence read depths would have been required to identify HIV-1. In addition, more specialist approaches are required to reliably identify HIV-1 and assembly genomes and rely on preamplification (34). The evidence suggested potential activation of HHV4, and this could contribute to T cell dysfunction in these patients and, as noted, immune cell subsets may have different frequencies in different patients with EVD.

The deep sequencing and bioinformatics approaches showed that nucleic acids from viral, bacterial, fungal, and parasitic organisms were present in the blood taken from many patients. Many of the potentially identified bacteria are associated with sepsis and consideration should be given to treating patients with antibiotic therapy. While *Plasmodium* spp. can be detected by the applied rapid test under field conditions, the deep sequencing data suggest that this approach did not detect normal parasite carriage in the EBOV-infected patient population, and this may have affect on lethality calculations.

This is the first study to demonstrate that a potential range of pathogens are present in the blood of patients with EVD and provide evidence for existing treatment guidelines (antimicrobial therapy). The study provides a comprehensive open access data set for researchers to investigate a range of microbial factors linked to EBOV pathogenesis and outcome. Although we have learned much about the clinical syndrome of EVD and natural history of the disease, the pathogenesis of disease is still not understood. Much of the research focus focused on the 2013–2016 outbreak has been with regard to developing clinical trials for EBOV-specific therapeutics, but little evidence exists for different supportive care platforms, including empirical antimicrobial use. Deep sequencing of historical samples from patients can help fill this vital knowledge gap and illustrates research requirements for future EBOV outbreaks, mainly to build in prospective, preapproved protocols for EVD with serial sampling, matched clinical data, and correlation with other standardized microbiological techniques.

## MATERIALS AND METHODS

### Patients.

Four main categories of patients were selected for analysis (Table 1). In the first two groups, RNA was extracted from (mainly) blood or plasma samples, taken from acutely ill patients during assessment of potential EVD cases, by the European Mobile Laboratory, located in the Médecins Sans Frontières (MSF) Ebola Treatment Centre Guéckédou in Guinea. These patients either went on to survive (hospitalized survivors, *n* = 44) or had a fatal infection (hospitalized fatalities, *n* = 118). These patients were also tested for *Plasmodium*. For the latter analysis, noninactivated EDTA blood was applied to an immunochromatographic assay (BinaxNOW Malaria; Alere GmbH, Cologne, Germany). These acute patients were treated according to MSF guidelines. In the third category, RNA was prepared from blood samples taken from patients who were convalescent for EVD and were qRT-PCR negative for EBOV (convalescent controls, *n* = 16). In a fourth group, RNA was extracted from oral and throat swabs taken from people who had already died of EVD (community deaths, *n* = 24). This last group was selected to evaluate microbiome sampling using swabs, as these are a suggested alternative to peripheral blood for determining EBOV status or an alternative to cardiac puncture blood for deceased patients. All RNA was stored at −20°C until shipment.

The National Committee of Ethics in Medical Research of Guinea approved the use of diagnostic leftover samples and corresponding patient data for this study (permit number 11/CNERS/14). Ethical permission for the sequencing work conducted at the University of Liverpool on RNA from patient samples was reviewed and approved by the institution under reference number RETH000784. Permission to sequence biological samples (made safe) containing genetic material from human pathogen hazard group 4 viruses was granted by the UK Home Office and the United Kingdom National Counter Terrorism Security Office. Ethical permission was also obtained from the Ethik-Kommission Der Arztekammer Hamburg (PV4910). Experimental methods complied with the Helsinki Declaration. For samples obtained from community deaths, hospitalized survivors, and hospitalized fatalities, these samples were collected as part of the public health response to contain the outbreak in Guinea, and informed consent was not obtained from these patients. For samples from convalescent patients, informed consent was obtained.

There was no significant difference between onset of symptoms (as reported by the patient) and sample procurement for admittance into the Ebola Treatment Centre (Fig. 1).

### RNA sequencing and bioinformatics.

Extracted RNA was DNase treated and sequenced using a HiSeq2500 system, with no preamplification of any sequence, as described elsewhere (2, 35) and reproduced herein. Samples from patients were sequenced on a HiSeq2500 system, and several criteria were applied to the selection of data postsequencing. The RNA was DNase treated using Ambion Turbo DNase. RNA-seq libraries were prepared from the DNase-treated RNA by using the Epicentre ScriptSeq v2 RNA-seq library preparation kit and performing 10 to 15 cycles of amplification. Libraries were purified using AMPure XP beads. Each library was quantified using Qubit, the size distribution was assessed using the Agilent 2100 Bioanalyser, and the final libraries were pooled in equimolar ratios. The quantity and quality of each pool were assessed with the Bioanalyzer and subsequently by qPCR using the Illumina library quantification kit from Kapa on a Roche Light Cycler LC480II system according to the manufacturer’s instructions. The template DNA was denatured according to the protocol described in the Illumina User Guide and loaded at 12 pM. To improve sequencing quality control, samples were spiked with 1% PhiX. The sequencing was undertaken on the Illumina HiSeq 2500 with version 4 chemistry, generating 2- by 125-bp paired-end reads. Base calling and demultiplexing of indexed reads were performed by using Casava version 1.8.2 (Illumina) to produce all the sequence data in fastq format. The raw fastq files were trimmed to remove Illumina adapter sequences by using Cutadapt version 1.2.1. The option -O 3′ was set, so that the 3′ ends of any reads which matched the adapter sequence over at least 3 bp were trimmed off. The reads were further trimmed to remove low-quality bases, using Sickle version 1.200 with a minimum window quality score of 20. After trimming, reads shorter than 10 bp were removed. Resulting sequence reads were initially mapped to the human genome using the short-read mapper Bowtie2 as previously described (2). Those that did not map to the human genome were then pooled and mapped again to a list of known human transcripts again using Bowtie2. Sequences that did not align to either the human genome or human transcriptome were pooled, but the size of the data set (~1 TB of fastq reads) required them to be preprocessed first by the Trinity read normalization software to reduce the memory requirements and runtimes prior to *de novo* assembly using Trinity (36). The assembled data from Trinity were first manually checked to determine that the EBOV genome had been assembled before all the reads were checked for matches using Diamond BLASTX analysis against the nonredundant protein database (37). Vertebrate BLASTX hits, including some for several nonhuman primates, were excluded, as these were deemed likely high-copy human sequences which had passed through the previous filters. Thirty-two contigs with a top hit to *Medicago sativa* (alfalfa) were also excluded, as these had a taxonomically diverse array of matches with similar E values, indicative of spurious annotation. For each transcript, the best hit was retained and the protein name and organism for that hit was associated with the transcript using in-house scripts. Quality and primer trimmed reads were used as input for MetaPhlAn 2 for estimating genome coverage of bacterial species for each sample. In general, for viral species a 20% mapping to a transcriptome was adopted as a threshold for positive identification.

### Mass spectrometry analysis.

Selected EBOV-positive samples underwent bacterial cultivation under biosafety level 4 conditions, including samples of whole blood and throat swabs. The samples were inoculated into bouillon broth enriched with 10% fetal bovine serum, incubated at 37°C in the presence of 5% CO_2_, and after 24 h, 48 h, and 72 h the samples were plated onto blood agar. The mixed cultures were isolated and underwent three subculture passages on blood agar. Colonies were identified from throat swabs, but not from peripheral blood samples, and the colony samples were analyzed by MALDI-TOF (Bruker MALDI Biotyper).

### Accession number(s).

Raw sequence read data for all the samples used in this analysis were deposited with NCBI, BioProject ID PRJNA352396. Note that the data contain no patient-identifiable information, only the outcome of infection.
