# Supplementary figures and images for: Deep Sequencing of RNA from Blood and Oral Swab Samples Reveals the Presence of Nucleic Acid from a Number of Pathogens in Patients with Acute Ebola Virus Disease and Is Consistent with Bacterial Translocation across the Gut
Source: mSphere. 2017 Aug 23;2(4):e00325-17. doi: 10.1128/mSphereDirect.00325-17 (PMC5566839; doi:10.1128/mSphereDirect.00325-17)

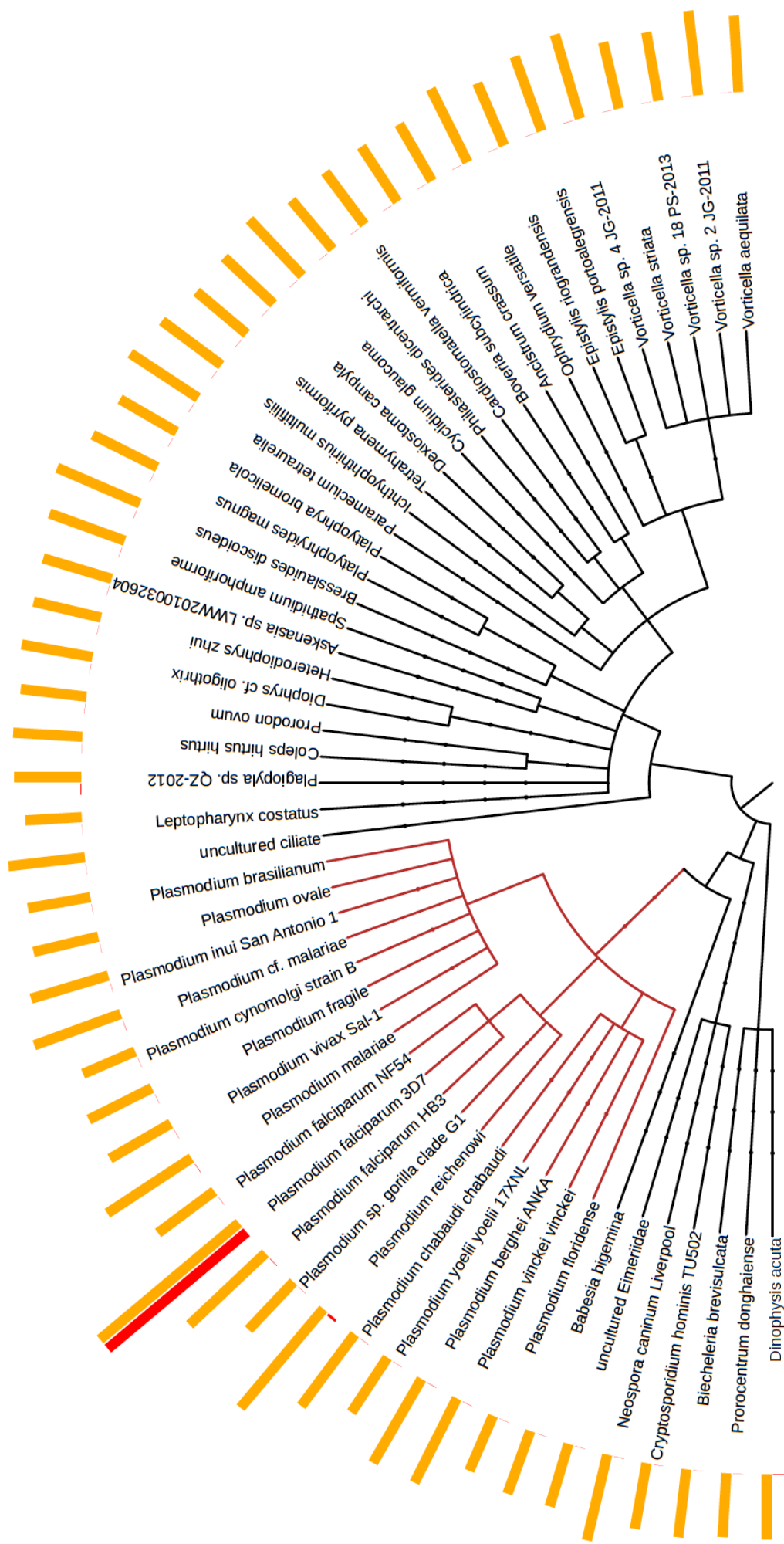

Supplement: FIG S1 [file sph004172346sf2.pdf]
